# Miniaturised Extraction Techniques in Personalised Medicine: Analytical Opportunities and Translational Perspectives

**DOI:** 10.3390/molecules30214263

**Published:** 2025-10-31

**Authors:** Luana M. Rosendo, Tiago Rosado, Mário Barroso, Eugenia Gallardo

**Affiliations:** 1 RISE-Health, Departamento de Ciências Médicas, Faculdade de Ciências da Saúde, Universidade da Beira Interior, 6201-506 Covilhã, Portugal; may.rosendo@ubi.pt; 2 Laboratório de Fármaco-Toxicologia, Universidade da Beira Interior, 6201-000 Covilhã, Portugal; 3 Grupo de Problemas Relacionados com Toxicofilias, Centro Académico Clínico das Beiras (CACB), 6200-000 Covilhã, Portugal; 4 Serviço de Química e Toxicologia Forenses, Instituto Nacional de Medicina Legal e Ciências Forenses-Delegação do Sul, 1169-201 Lisboa, Portugal; 5 Alpha Biolabs, 14 Webster Court, Carina Park, Westbrook, Warrington WA5 8WD, UK

**Keywords:** miniaturised extraction techniques, bioanalysis, drug monitoring, validation, biological fluids

## Abstract

Miniaturised sampling and extraction are redefining therapeutic drug monitoring (TDM) by enabling low-volume sampling, simplifying collection, and improving patient acceptability, while also promoting decentralised workflows and more sustainable laboratory practices. This review critically appraises the current landscape, with emphasis on analytical performance, matrix compatibility, and readiness for clinical implementation. It examines validation requirements, the extent of alignment and existing gaps across major regulatory guidelines, and recurrent challenges such as haematocrit bias, real-world stability and transport, incurred sample reanalysis, device variability, commutability with conventional matrices, and inter-laboratory reproducibility. To make the evidence actionable, operational recommendations are distilled into a practical ten-point checklist designed to support validation and translation of miniaturised approaches into routine laboratory practice. Looking ahead, priorities include automation and portable platforms, advanced functional materials, and integration with digital tools and biosensors, alongside the development of harmonised frameworks tailored to miniaturised methods and prospective clinical studies that demonstrate impact on dosing decisions, adherence, and clinical outcomes. Overall, this review aims to equip researchers, laboratory professionals, and regulators with the knowledge to implement miniaturised bioanalysis and advance personalised medicine through TDM.

## 1. Introduction

Over the past two decades, personalised medicine has markedly shifted the paradigm of pharmacotherapy, aiming to tailor treatment strategies to the genetic, physiological, and environmental profiles of each individual [1,2]. Central to this approach is the need for accurate and timely information on drug levels and pharmacokinetics in specific patients, particularly in the context of therapeutic drug monitoring (TDM), dose individualisation, and the early detection of adverse effects [1,3,4].

This shift has placed increasing demands on bioanalytical chemistry, particularly with respect to sample collection, preparation, and analysis [5,6,7]. Traditional workflows in clinical laboratories, which typically rely on venous blood collection, centrifugation, and multi-step sample processing, are not always compatible with the requirements of real-time, patient-centred monitoring [6,8,9]. These limitations are especially critical in vulnerable populations (e.g., paediatric, geriatric, or critically ill patients) as well as in remote or decentralised care settings, where conventional methods can become logistically and ethically challenging [8,10,11].

In response, miniaturised extraction techniques have emerged as powerful tools to enhance bioanalytical flexibility and robustness. These approaches are characterised by their low sample volume requirements, reduced solvent consumption, improved portability, and strong potential for integration into simplified or even automated workflows [5,6,9,12,13]. Techniques such as dried matrix spots (DMS), microextraction by packed sorbent (MEPS), solid-phase microextraction (SPME), and fabric phase sorptive extraction (FPSE) have already shown promising applications in clinical, toxicological, and forensic contexts [5,12,14,15]. More recently, emerging strategies—including micro solid-phase extraction (µSPE), miniaturised adaptations of Quick, Easy, Cheap, Effective, Rugged, and Safe (QuEChERS), and novel sorbent-based platforms—have further expanded the bioanalytical toolbox available to meet the demands of modern healthcare [15,16,17,18,19].

Despite the increasing number of studies exploring these techniques individually, a comprehensive and critical overview of their analytical performance, applicability to TDM and special populations, and real-world implementation challenges is still lacking [6,8]. Moreover, uncertainties remain regarding validation strategies, regulatory acceptance, and inter-laboratory reproducibility of these miniaturised methods, limiting their wider adoption in regulated environments [8].

Miniaturised extraction techniques are thus increasingly recognised as essential tools for meeting the evolving analytical requirements of personalised medicine [5,6,8,9]. Their performance must be evaluated against specific technical needs, including those associated with therapeutic drug monitoring, sampling from alternative matrices, and remote or decentralised collection models [6,8,12,15]. Particular attention should be given to their comparative analytical performance, integration with advanced detection systems, and the regulatory and validation frameworks that govern their implementation [6,9,12,15]. A structured and critical perspective on these aspects is essential to establish their current role and to delineate their future potential in advancing personalised bioanalysis.

## 2. Analytical Requirements in Personalised Medicine

As personalised medicine becomes increasingly integrated into clinical practice, it brings with it a set of analytical demands that challenge conventional bioanalytical workflows [20]. The ability to deliver rapid, precise, and individualised data depends not only on the sensitivity and selectivity of the analytical method but also on the suitability of the sample collection and preparation strategy [21]. This section discusses the key analytical requirements associated with TDM, the specific needs of vulnerable or special populations, and the practical challenges of remote and home-based sampling approaches [1,8,9,14,20,22].

### 2.1. Therapeutic Drug Monitoring (TDM)

TDM plays a pivotal role in the individualisation of pharmacotherapy, particularly for drugs characterised by narrow therapeutic windows, marked pharmacokinetic variability, or a well-established relationship between plasma concentration and clinical response [1,5,8,14,22]. By enabling dose optimisation based on measured drug levels, TDM contributes directly to improved efficacy, reduced toxicity, and better clinical outcomes, which are central goals of personalised medicine [1,8,9,22].

From an analytical perspective, methods supporting TDM must provide highly accurate and reproducible results within clinically relevant timeframes. High sensitivity and selectivity are essential to reliably quantify drugs at therapeutic and subtherapeutic levels, often in complex biological matrices such as plasma or whole blood. Precision, accuracy, and reproducibility must be maintained across the validated therapeutic range, supported by robust calibration models and rigorous quality control protocols. The lower limit of quantification (LLOQ) must be sufficient to detect concentrations relevant for clinical decision-making [3,21,23,24,25].

The growing emphasis on minimally invasive strategies further increases the need for compatibility with low sample volumes, particularly in paediatric, geriatric, or critically ill patients [6,8,14]. Analytical stability during sampling, transport, and storage also becomes crucial, especially in decentralised or outpatient care settings [6,8].

TDM often requires frequent sampling to capture pharmacokinetic profiles, steady-state levels, or time-dependent variations. This highlights the need for minimally invasive or non-invasive sampling strategies, particularly in vulnerable populations such as neonates, paediatric patients, the elderly, and critically ill individuals, where conventional venepuncture may be impractical or ethically challenging [6,8,14,22].

Miniaturised extraction techniques address many of these limitations by enabling reliable analysis from small sample volumes, with simplified workflows and reduced invasiveness. They are particularly well-suited to decentralised, outpatient, or home-based contexts [8,9]. Their use is especially relevant for drugs requiring close monitoring—such as opioids, immunosuppressants, and antiepileptic agents—where therapeutic ranges are narrow and patient response highly individualised [10,26,27].

Miniaturisation also enhances sample stability during collection, transport, and storage, critical factors in remote care settings. The integration of miniaturised techniques thus represents a strategic step in aligning analytical capabilities with the clinical demands of TDM in personalised medicine [6,28,29].

### 2.2. Bioanalysis in Vulnerable and Special Populations

Bioanalytical strategies for vulnerable populations require specific adaptations to ensure safety, accuracy, and feasibility. These groups—including neonates, infants, pregnant women, the elderly, and patients with chronic or acute medical conditions—often present ethical and physiological constraints that limit the use of standard sampling procedures such as venepuncture [6,8,30].

In many cases, the collection of large sample volumes is either impractical or clinically contraindicated due to low circulating blood volume, fragile health status, or increased risk of complications [6,8]. For instance, in neonatology, strict regulations limit the allowable blood volume for diagnostic purposes, and repeated sampling poses significant clinical risks [11]. Similarly, in patients with cognitive or physical impairments, conventional sampling protocols may be poorly tolerated or unfeasible, necessitating simplified procedures with minimal intervention [30].

The frequent monitoring required in these populations underscores the importance of minimally invasive and low-volume approaches. Ethically, reducing patient burden and discomfort is paramount, particularly in paediatric and geriatric contexts [6,28,30]. Logistically, these groups are often managed in outpatient, home-based, or intensive care settings, where conventional laboratory workflows may not be feasible [8,28].

The increasing use of decentralised sampling, including remote and home-based collection, introduces further analytical demands. Ensuring sample stability after collection—particularly during drying, storage, and transport—is essential to prevent analyte degradation. In addition, maintaining traceability and chain of custody is critical when samples are collected outside controlled clinical environments [6,30].

From a bioanalytical perspective, sample preparation is decisive for workflow success. Techniques must be compatible with small volumes, simplify matrix complexity, and provide consistent analyte recovery under variable pre-analytical conditions. Miniaturised extraction strategies, including DMS formats and other microscale approaches, offer practical and robust solutions that meet these requirements while supporting the broader goals of personalised and decentralised medicine [6,8,30].

### 2.3. Remote and Home-Based Sampling

Remote and home-based sampling strategies are becoming increasingly integrated into healthcare systems, particularly in the management of chronic disease, decentralised clinical trials, and digital health platforms [31,32,33]. These models offer significant advantages in terms of patient autonomy, accessibility, and longitudinal monitoring, while reducing pressure on healthcare infrastructure [34].

However, they also introduce analytical challenges that must be overcome to ensure data quality, reproducibility, and regulatory compliance [35]. Sampling procedures must be simple enough to allow self-collection or caregiver-assisted collection, while minimising the risk of errors [36]. In addition, sample integrity must be preserved during transport—often under ambient conditions and without access to cold-chain logistics—which may affect analyte stability, particularly for labile compounds [14,37].

Samples collected in remote contexts must also be fully compatible with downstream analytical methods, such as liquid chromatography–mass spectrometry (LC-MS/MS), without introducing variability due to degradation, contamination, or volume inconsistency [38,39,40]. These requirements emphasise the need for robust and standardised sampling formats that can ensure accurate, reproducible bioanalysis outside clinical laboratories [30].

Miniaturised extraction techniques, especially dried formats such as dried blood spots (DBS) and dried saliva spots (DSS), are particularly suited to remote and home-based applications [14,28,38]. These approaches provide advantages such as improved analyte stability, portability, and reduced biohazard risk, supporting safe and effective sample handling beyond conventional clinical environments [14,30]. As a result, they have become integral components of bioanalytical strategies in personalised medicine, enabling flexible and scalable therapeutic monitoring in decentralised healthcare [1,30].

## 3. Miniaturised Extraction Techniques and Their Analytical Suitability

As highlighted in the previous sections, the shift towards decentralised, low-volume, and patient-centred sampling strategies in personalised medicine introduces specific demands for sample preparation workflows [6,8,9]. Miniaturised extraction techniques have therefore emerged as key innovations in modern bioanalysis, enabling efficient and robust processing of microsamples while maintaining analytical performance [5,6,8,9].

The development of these methods has been driven by the need to reduce sample volume, solvent consumption, and turnaround time without compromising sensitivity, selectivity, or reproducibility. Importantly, they are aligned with the principles of green analytical chemistry and provide practical benefits in clinical and translational settings, particularly when remote or longitudinal sampling is required [6,8,9].

A distinctive feature of miniaturised extraction is the ability to isolate analytes directly from microsamples, often ≤100 µL, using compact and efficient formats [5,6,8,9]. These techniques typically rely on sorbent-based mechanisms with high surface area and strong analyte retention, facilitating selective pre-concentration and clean-up [5]. Several sorbent-based miniaturised formats have been developed over the past decade, each optimised for different matrices and analytical requirements, as detailed in the following subsections [5,6,7,12,16,19]. Their compatibility with LC-MS/MS, GC-MS, and automated workflows makes them particularly suited to TDM and other high-throughput applications [5,8,9,40].

To support a critical analysis of the current state of miniaturised extraction techniques in TDM, a targeted literature search was conducted using PubMed and Scopus. The general inclusion period was set from January 2020 to May 2025 to capture recent methodological advances and translational trends. However, an extended range was applied for MEPS (2015–2025) and FPSE (2018–2025), reflecting their earlier emergence and wider application history. This adjustment ensured the inclusion of representative and validated methods relevant for comparative analysis. The search strategy combined keywords such as *miniaturised techniques*, *microextraction*, *therapeutic drug monitoring*, *TDM*, *biological matrices*, *microextraction by packed sorbent*, *solid-phase microextraction*, *dried matrix spots*, *fabric phase sorptive extraction*, *dispersive liquid–liquid microextraction*, and *human*. Filters were applied to exclude reviews, conference abstracts, and studies without drug quantification in biological samples. Only original articles presenting validated analytical data and detailed methodological descriptions were included. The resulting studies are summarised and critically discussed in the subsections below.

### 3.1. Dried Matrix Spots (DMS)

DMS techniques involve depositing small volumes of biological samples onto porous substrates, followed by ambient drying and subsequent extraction [8,9,28]. DBS is the most established format, widely used in neonatal screening and TDM [8,14,30]. DSS and DPS extend this concept to oral fluid and plasma, enabling non-invasive or matrix-specific applications [8,41,42].

The main analytical advantages of DMS include enhanced analyte stability at room temperature, simplified transport logistics, and compatibility with very low sample volumes (as little as 10–30 µL) [6,28,43]. These characteristics make DMS particularly attractive for TDM in paediatric populations and decentralised settings. For example, tacrolimus and antiepileptics have been successfully quantified from DBS in transplant patients, with clinically acceptable agreement compared to venous blood sampling [26,44,45]. Representative applications of DMS across different matrices and therapeutic classes are summarised in Table 1.

Nonetheless, important limitations remain. In DBS, haematocrit-related variability can affect analyte diffusion and quantification. Other issues include uneven analyte distribution across the spot and reduced recovery of highly hydrophilic compounds. Consequently, pre-validation of drying time, spot homogeneity, and elution efficiency is essential to ensure method standardisation and clinical reliability [28,30,38].

Overall, Table 1 illustrates the broad versatility of DMS across therapeutic areas. DBS is the most widely implemented format, particularly for immunosuppressants and antiepileptics, with both traditional filter cards and novel devices such as HemaPEN^®^, HemaXis^®^ DB10, and Capitainer^®^ showing clear clinical utility [44,47,54,64]. In addition, clozapine, olanzapine, and risperidone have been successfully quantified in psychiatric settings using automated DBS-SPE-LC-MS/MS workflows [48].

In brief, DMS is effective for TDM (notably via DBS), while haematocrit effects, spot heterogeneity and recovery of hydrophilic compounds remain the principal constraints.

### 3.2. Microextraction by Packed Sorbent (MEPS)

MEPS is a miniaturised adaptation of traditional solid-phase extraction (SPE), in which the sorbent material is integrated directly into a syringe or cartridge. During operation, the sample is aspirated and dispensed repeatedly through the sorbent bed, enhancing analyte–sorbent interactions and retention, followed by elution with microlitre volumes of solvent [69,70].

MEPS is applicable to a wide range of drugs and biological matrices and has been extensively employed for the monitoring of antidepressants, opioids, and anticancer agents [71,72,73,74]. Its compact format and suitability for automation make it particularly attractive for routine TDM, both in centralised laboratories and in bedside applications with robotic samplers [7,75]. Direct coupling of MEPS to LC-MS/MS further reduces turnaround time, a key advantage in dose adjustment protocols [7]. Representative examples of MEPS applications across bioanalytical contexts, including target analytes, matrices, and clinical relevance, are summarised in Table 2.

MEPS accommodates sub-100 µL samples, useful in paediatric/geriatric settings, provided that sorbent conditioning, carryover control and cartridge reuse are rigorously standardised to ensure reproducibility and compliance [69,75].

MEPS has been applied to plasma, oral fluid, urine, serum, and whole blood for the determination of a wide variety of therapeutic drugs. Reported applications include the quantification of multiple psychoactive compounds (e.g., fluoxetine, haloperidol, clomipramine, mirtazapine) in plasma from patients with schizophrenia [71], antipsychotics with RACNT sorbent [76], ziprasidone [77], antiepileptics such as zonisamide [78] and carbamazepine/lamotrigine [71], antidepressants (venlafaxine and *O*-desmethylvenlafaxine) [75], the antiarrhythmic amiodarone [80], local anaesthetics (lidocaine, ropivacaine, bupivacaine) [82], immunosuppressants (cyclosporine A, everolimus, mycophenolic acid, sirolimus, tacrolimus) [81], and opioids such as methadone, morphine and buprenorphine in oral fluid [74].

The main advantages of MEPS include reduced solvent consumption, short analysis time, and strong potential for automation, making it highly suitable for high-throughput laboratories [13,75]. However, careful handling is required to minimise carryover and prevent sorbent degradation. Furthermore, its dependence on laboratory infrastructure limits its applicability in remote or patient-led sampling. Despite these constraints, MEPS remains a robust and practical tool for routine TDM in centralised facilities [5,75].

### 3.3. Solid-Phase Microextraction (SPME)

SPME is primarily a solvent-free technique that employs a coated fibre or capillary to extract analytes from biological samples through adsorption or absorption mechanisms, followed by thermal or, when required, solvent desorption [13,16,83,84]. In-tube SPME, a more robust and automation-compatible variant, can be directly integrated with chromatographic systems, making it particularly suitable for complex biological matrices [16,19,84,85].

Like MEPS and FPSE, SPME is a sorbent-based miniaturised extraction approach derived from SPE, sharing the same fundamental retention principle while differing in device configuration and extraction dynamics [5,6,7,12,16].

SPME has shown considerable potential in the bioanalysis of volatile and semi-volatile compounds, including benzodiazepines and certain antipsychotics [83]. It offers high enrichment factors, minimal matrix effects, and eliminates the use of organic solvents, aligning well with the principles of green analytical chemistry [16,83,84]. Within personalised medicine, SPME has been successfully employed for longitudinal drug monitoring and metabolite profiling in plasma and oral fluid samples [5,83].

Despite these advantages, the use of SPME in aqueous biological fluids often requires careful optimisation of coating selectivity to improve analyte retention and reproducibility. Additionally, the fragility of extraction devices remains a limitation in high-throughput or decentralised settings, unless mitigated by automation or protective hardware [84].

Representative applications are listed in Table 3.

SPME has demonstrated strong potential in TDM across diverse drug classes. Reported applications include the determination of antidepressants, antipsychotics, and antiepileptics (e.g., amitriptyline, venlafaxine, quetiapine, aripiprazole, lamotrigine, carbamazepine) in whole blood from psychiatric patients [83], immunosuppressants such as tacrolimus, sirolimus, everolimus and cyclosporine A in transplant settings [86]; and anticancer agents, including gefitinib [87] and imatinib [88]. Intraoperative and near real-time monitoring has been demonstrated for tranexamic acid in plasma and urine [89,92]. Other notable applications include the monitoring of levodopa and related metabolites in Parkinson’s disease [90]; free plasma concentrations of drugs with diverse physicochemical properties, such as atenolol, morphine, lorazepam, and buprenorphine [91]; caffeine in neonatal therapy [93]; valproic acid in serum [94]; and tricyclic antidepressants in serum and tissues using covalent organic frameworks [95]. SPME has also been extended to tissue analysis, enabling the quantification of doxorubicin and its metabolites in lung samples [96].

Recent developments have expanded SPME’s analytical capability through direct coupling with ambient ionisation mass spectrometry (MS) sources, such as direct analysis in real time (DART-MS), desorption electrospray ionisation (DESI-MS), and paper spray MS (PS-MS). These configurations eliminate the chromatographic step, enabling rapid, solvent-free analysis directly from the extraction device. For example, BioSPME fibres coated with hydrophilic–lipophilic balance (HLB) particles have been successfully interfaced with microfluidic open interface MS/MS systems (MOI-MS/MS) for real-time quantification of immunosuppressants [86,89]. Similarly, ambient SPME–MS platforms have been applied to tranexamic acid in plasma and urine [89,92], and to tricyclic antidepressants in biological tissues using electrospray ionisation (ESI-MS) [95]. These hybrid approaches offer high-throughput capability and minimal sample handling, providing a promising route toward point-of-care or bedside TDM applications. Although still at an early developmental stage, SPME–ambient MS coupling represents a major step toward rapid, portable, and minimally invasive bioanalysis.

The technique offers high enrichment capacity, minimal matrix effects, and compatibility with automated LC-MS/MS workflows, making it particularly attractive for clinical and pharmacokinetic applications. Nonetheless, limitations such as fibre fragility, variability in coating reproducibility, and the need for specialised desorption equipment remain challenges to its broader routine implementation.

### 3.4. Fabric Phase Sorptive Extraction (FPSE)

FPSE is a relatively recent miniaturised technique that integrates a porous fabric substrate with a chemically bonded sorbent coating, representing an evolution of SPE principles into a flexible, fabric-supported format [5,6,7,12,16].

This configuration produces a durable, flexible, and high-capacity extraction device that can be directly immersed in biological fluids, thereby simplifying workflows and reducing the need for extensive sample pre-treatment [97,98].

FPSE has been applied to therapeutically relevant compounds in matrices suited to TDM, including oral fluid, plasma, and urine. Reported targets encompass antidiabetics and insulin secretagogues, xanthine-oxidase and leukotriene-receptor modulators, antivirals, and antidepressants, among others [97,99,100,101,102,103]. These attributes support less invasive and decentralised sampling strategies—such as oral-fluid-based monitoring—that align with patient-centred TDM workflows [97,99,100,101,102].

Despite its promise, FPSE remains at an early stage in terms of regulatory integration. Wider adoption will require inter-laboratory studies, protocol harmonisation, and explicit alignment with established bioanalytical validation frameworks (FDA, EMA/ICH M10) [21,23,24,25]. Selected bioanalytical applications relevant to personalised medicine are summarised in Table 4.

FPSE has been successfully applied to a range of therapeutic classes, including antidiabetic agents such as pioglitazone, repaglinide, and nateglinide in plasma [99]; febuxostat and montelukast for pharmacokinetic profiling [100]; and the antiviral favipiravir in both plasma and breast milk [101]. It has also been employed for the determination of ciprofloxacin, sulfasalazine, and cortisone in plasma, blood, and urine [97], as well as multiple antidepressants (venlafaxine, citalopram, paroxetine, fluoxetine, sertraline, amitriptyline and clomipramine) across blood, urine and oral fluid [102]. Beyond pharmaceuticals, FPSE has enabled the biomonitoring of UV filters and benzophenone derivatives in human samples [98]. More recently, separation and enrichment of fingolimod and citalopram have been demonstrated in artificial matrices relevant to multiple sclerosis therapy [103].

Collectively, these studies highlight the versatility of FPSE, driven by the tunable polarity and selectivity of sol–gel chemistries, as well as its compatibility with LC–MS/MS and HPLC workflows. Nonetheless, the transition towards clinical routine requires broader inter-laboratory validation, standardisation of protocols, and demonstration of regulatory compliance.

### 3.5. Dispersive Liquid–Liquid Microextraction (DLLME)

DLLME is a rapid, solvent-minimising extraction approach in which a disperser solvent delivers a small volume of extractant into the aqueous sample, generating fine droplets that maximise interfacial area and accelerate analyte partitioning. The technique provides high enrichment factors with short equilibration times and can be readily coupled with GC–MS, HPLC, or LC–MS/MS workflows [29]. Variants such as ultrasound-assisted DLLME and deep eutectic solvent (DES)-based DLLME further extend applicability while improving sustainability profiles [104,105,106].

In bioanalytical and TDM-focused studies, DLLME has been applied across diverse drug classes and clinical matrices, supporting pharmacokinetic assessment and therapeutic monitoring in real patient samples (Table 5) [106,107,108,109,110,111]. While the method typically requires centrifugation and benchtop infrastructure, its speed, selectivity for hydrophobic analytes, and very low solvent consumption make it a versatile and attractive option for routine laboratory settings [29,107,108,109].

Representative applications include anticancer agents such as letrozole, anastrozole, palbociclib, ribociclib, abemaciclib, and fulvestrant in plasma from breast cancer patients [112,114]; tamoxifen and its metabolites (*N*-desmethyltamoxifen, 4-hydroxytamoxifen, endoxifen) [109]; and docetaxel in paediatric oncology [111]. The technique has also been employed for psychiatric and neurological drugs, including chlorpromazine [113]; risperidone and 9-hydroxyrisperidone [108]; carbamazepine and phenobarbital [110]; and antidepressants such as amitriptyline, imipramine, sertraline and fluoxetine using deep eutectic solvents [106]. In infectious disease and immunosuppressive therapy, DLLME has been used to efavirenz [107] and sirolimus in blood samples from paediatric patients [115]. Across these applications, DLLME demonstrates high extraction efficiency, extremely low solvent consumption, and broad compatibility with diverse drug classes and detection techniques (GC–MS, HPLC, LC–MS/MS). Variants such as ultrasound-assisted DLLME (UA-DLLME) and deep eutectic solvent-based DLLME (DES-DLLME) have further extended applicability while improving the sustainability profile [106,111]. However, DLLME typically requires centrifugation and benchtop laboratory infrastructure, which limits its suitability for decentralised or point-of-care testing. Despite these constraints, DLLME remains a versatile and powerful tool for the selective enrichment of hydrophobic drugs in clinical bioanalysis.

### 3.6. Emerging Approaches

Recent advances in miniaturisation have stimulated the development of innovative extraction strategies designed to operate with microvolumes of biological samples [5,6,8]. These approaches aim not only to minimise solvent and sample requirements but also to expand analytical versatility through novel sorbent chemistries and device formats [5,7,29].

Among these, µSPE variants (including particulate- and monolith-based designs) have been applied to plasma, serum, and urine for the quantification of a wide range of therapeutic agents [5]. Volumetric absorptive microsampling (VAMS) has emerged as a user-friendly alternative to dried spot sampling, and is already in routine use for monitoring immunosuppressants and antidepressants [10,104]. In parallel, disposable pipette extraction (DPX) and dispersive solid-phase extraction (dSPE, m-µSPE) have demonstrated rapid, automation-compatible workflows [7,75,105].

Other promising innovations are based on engineered sorbent materials [7]. Thin-film microextraction (TFME) has been fabricated using molecularly imprinted polymers or biopolymers, such as polylactic acid, enabling the targeted enrichment of anticancer and antibiotic drugs. Electromembrane extraction (EME) and hollow-fibre liquid-phase microextraction (HF-LPME) allow highly selective partitioning of analytes across polymeric membranes, whereas salt-assisted liquid–liquid microextraction (SALLME) provides a simple and low-cost option for plasma and urine matrices [29,116,117]. Additionally, mini-QuEChERS adaptations have been investigated for psychotropic drugs in blood, extending the application of food safety methodologies to clinical and forensic contexts [1,104].

Although most of these techniques remain at the proof-of-concept or translational research stage, they show strong potential for TDM and pharmacokinetic studies. Their integration with high-resolution mass spectrometry, portable detectors, and point-of-care devices may ultimately facilitate more personalised and decentralised drug monitoring [8]. Table 6 summarises representative applications of these emerging approaches across different drug classes and biological matrices.

Collectively, these emerging approaches demonstrate the trend towards platform miniaturisation and advanced sorbent chemistries. VAMS, for example, is already used in routine clinical practice for sirolimus monitoring in paediatric transplant patients [126] and for antidepressants in blood and oral fluid [127]. µSPE has enabled the simultaneous determination of cannabidiol and tetrahydrocannabinol in plasma [92], while TFME using biopolymers or molecularly imprinted membranes has been applied to anticancer drugs such as dasatinib and erlotinib [132]. Electromembrane extraction (EME) has been tested for phenytoin in plasma using hollow fibres [134]. These techniques are attractive due to their compatibility with automation, portability, and green chemistry principles, but remain at an early translational stage, with limited large-scale validation or clinical outcome data.

Overall, these innovations mark a significant step towards greener, faster, and more integrated analytical workflows. Their continued development and validation will be crucial to enable consistent implementation in therapeutic drug monitoring and personalised medicine. The comparative performance of these emerging methods in relation to established miniaturised techniques is further discussed in Section 3.7.

### 3.7. General Discussion

The comparative analysis of miniaturised extraction techniques presented in Table 1, Table 2, Table 3, Table 4, Table 5 and Table 6 highlights their growing importance in TDM but also underscores marked differences in analytical performance, operational feasibility, and clinical readiness. Although all approaches aim to facilitate accurate drug quantification from microsamples, their suitability for routine application in personalised medicine remains variable.

Across the reviewed studies, LOQs varied considerably between extraction techniques for the same compounds, reflecting differences in matrix handling and enrichment efficiency. For antidepressants, sorbent-based miniaturised formats consistently achieved lower LOQs than solvent-based protocols. For instance, amitriptyline reached 0.08 ng/mL with MEPS [72], while SPME yielded 0.92 ng/mL [83] and 0.30 ng/mL [95]; by contrast, DLLME reported an LOQ of 0.075 μg/mL for the same analyte [106]. This clear difference underscores the superior matrix clean-up and preconcentration efficiency of sorbent phases for trace-level quantification in TDM. Similarly, for antipsychotics, particularly risperidone and its metabolite 9-hydroxyrisperidone, DBS/DMS workflows achieved LOQs of 0.22 ng/mL and 9.85 ng/mL, respectively [48], whereas DLLME yielded LOQs around 5 ng/mL [108]. These differences are analytically meaningful in TDM, where low-level quantification directly supports dose adjustment and assessment of therapeutic adherence. For immunosuppressants such as tacrolimus, MEPS achieved the lowest LOQ (0.113 ng/mL) [81], outperforming SPME (0.80 ng/mL) [86] and DMS (1.0 ng/mL) [44]. This hierarchy is consistent with the strong clinical requirement for sub-ng/mL sensitivity in transplant patient monitoring, highlighting the crucial role of miniaturised extraction techniques in precise immunosuppressant quantification.

Together, these examples illustrate how miniaturised extraction techniques differ not only in their operational characteristics but also in their achievable analytical sensitivity. Such comparisons reinforce the importance of selecting extraction methods strategically according to drug class, matrix, and clinical context to ensure reliable and sensitive therapeutic drug monitoring.

DMS techniques (Table 1) are by far the most widely employed, particularly in paediatric, transplant, and remote care contexts. Drugs such as tacrolimus, levetiracetam, and lamotrigine have been successfully quantified using DBS formats with strong correlation to venous plasma levels. For instance, tacrolimus has been monitored in transplant patients using HemaPEN^®^ devices [44], while levetiracetam and other antiepileptics were analysed using Whatman 903 cards [45], demonstrating both analytical sensitivity and logistical advantages. Similarly, antipsychotics, including olanzapine, clozapine, and risperidone, have been quantified with AutoCollect™ DBS cards in automated DBS–Online SPE–LC–MS/MS systems [48], underscoring adaptability to high-throughput workflows. Nonetheless, DMS remains strongly affected by haematocrit variability, spot homogeneity, and uneven analyte distribution, particularly for hydrophilic compounds. Despite improvements with advanced devices such as Capitainer^®^ and HemaXis^®^, full standardisation remains a challenge.

By contrast, MEPS (Table 2) enables precise and reproducible extraction under controlled conditions, particularly in plasma matrices. It has been applied to the quantification of antidepressants and antipsychotics such as fluoxetine, clomipramine, haloperidol, and mirtazapine in plasma from psychiatric patients [71]. In addition, opioids, including morphine, methadone, and buprenorphine, have been monitored in oral fluid using MEPS cartridges in substitution therapy programmes [74], reflecting versatility across clinical and forensic contexts. MEPS offers low solvent consumption and automation potential, yet operational complexity, carryover risk, and the need for trained personnel restrict its use in patient-led sampling or decentralised settings.

SPME (Table 3) has emerged as a solvent-free alternative with substantial clinical promise. Applications include quantification of antidepressants and antipsychotics (e.g., quetiapine, aripiprazole, duloxetine) in whole blood using C_18_ SPME–LC fibres [64] and measurement of immunosuppressants such as sirolimus and cyclosporine A using Bio-SPME fibres [86]. Plasma and urine-compatible coatings have also enabled near real-time intraoperative monitoring of tranexamic acid [89]. Despite this potential, SPME devices are fragile, and coating reproducibility can vary, requiring careful optimisation when applied to complex biological matrices.

FPSE (Table 4), though less established, offers flexibility due to its fabric-based design and tunable sol–gel coatings. Reported applications include the quantification of antidepressants such as venlafaxine and sertraline across oral fluid, urine, and blood [102], and antidiabetic agents such as pioglitazone and repaglinide in plasma [99]. FPSE supports direct immersion in biological fluids and decentralised workflows, but most studies remain proof-of-concept, with limited data on inter-laboratory reproducibility.

DLLME (Table 5) provides excellent pre-concentration efficiency for lipophilic compounds. Examples include the quantification of tamoxifen and its metabolites in plasma [109], and simultaneous analysis of antidepressants such as fluoxetine and sertraline in plasma and urine using DES-assisted DLLME [106]. DLLME combines rapid extraction, low solvent use, and broad applicability; however, it still relies on centrifugation and benchtop infrastructure, which limits its suitability for point-of-care or home-based contexts.

Collectively, miniaturised extraction techniques represent complementary rather than competing strategies for bioanalysis in TDM. Sorbent-based methods, such as DMS, VAMS, MEPS, SPME, and FPSE, are generally closer to clinical translation due to their alignment with decentralised and patient-centred workflows.

**Figure 1:**
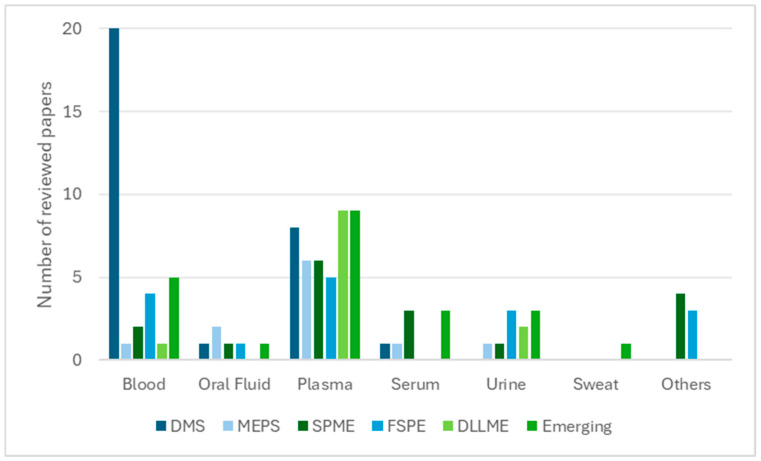
Relationship between biological matrices and extraction techniques (number of reviewed papers).

In contrast, solvent-based techniques such as DLLME, SALLME, HF-LPME, and EME remain predominantly laboratory-centred, offering superior enrichment for lipophilic compounds but limited applicability in remote or point-of-care settings. Together, these techniques illustrate the broader analytical transition towards automation, sustainability, and clinical readiness [109]. Matrix compatibility further distinguishes these approaches. Sorbent-based methods have been successfully extended to alternative matrices such as oral fluid, sweat, and breast milk, thereby broadening their clinical and forensic applicability. In contrast, solvent-based techniques remain largely confined to plasma and serum, where preparation conditions can be more rigorously controlled. This distribution is illustrated in Figure 1, showing that plasma and serum dominate solvent-based workflows, whereas sorbent-based methods display broader applicability to matrices such as oral fluid, urine, and sweat.

**Figure 2:**
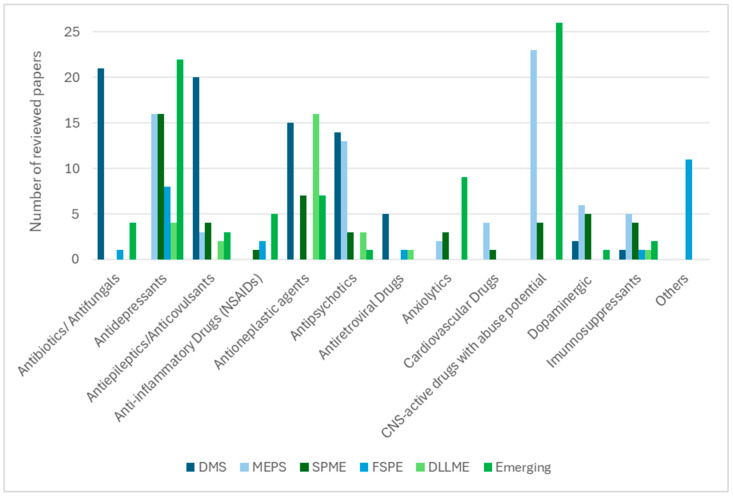
Distribution of drug classes analysed according to the extraction technique (based on the number of reviewed papers).

Sustainability and throughput also play decisive roles. VAMS and DBS reduce cold-chain logistics and facilitate remote monitoring [8,14,28,30,38], while greener DLLME variants employing deep eutectic solvents minimise environmental impact without compromising extraction efficiency [109]. MEPS and SPME stand out for their compatibility with automation, supporting high-throughput LC–MS(/MS) workflows [16,75,91]. The diversity of therapeutic classes analysed is summarised in Figure 2, where sorbent-based techniques are shown to be widely applied to antibiotics, antifungals, antidepressants, and CNS-active drugs, while solvent-based protocols are more frequently used for lipophilic drug classes such as antineoplastic and cardiovascular agents.

Overall, sorbent-based approaches appear closer to routine implementation in personalised medicine owing to their flexibility, patient-centred sampling, and readiness for automation. Conversely, solvent-based protocols remain indispensable for laboratory-based pre-concentration of challenging lipophilic compounds [5,6,8,10,14,16,30,91]. Rather than competing, these approaches form a complementary toolbox: sorbent-based techniques are best suited where decentralisation and patient convenience are priorities, while solvent-based methods continue to deliver high analytical sensitivity under controlled laboratory conditions [7,12,18,21,29,106,117]. Strategic selection of extraction methods according to drug class, biological matrix, and clinical context will therefore be essential to align analytical innovation with the evolving requirements of TDM [1,2,3,20].

Ultimately, the broader integration of miniaturised extraction techniques into personalised medicine will depend on harmonised validation protocols, robust inter-laboratory reproducibility, and demonstration of clinical equivalence. The studies summarised in Table 1, Table 2, Table 3, Table 4, Table 5 and Table 6 illustrate substantial progress, yet also emphasise the pressing need to bridge analytical innovation with regulatory alignment and practical clinical implementation [23,24,25].

## 4. Regulatory Considerations and Validation Challenges

Despite significant technological advances in miniaturised extraction and sampling techniques, their translation into routine clinical practice for TDM remains limited [28,30]. This is primarily due to concerns regarding regulatory compliance, inconsistent validation strategies, and limited inter-laboratory reproducibility.

Major agencies have set regulatory expectations for bioanalytical procedures, although these are only partially harmonised. The FDA Bioanalytical Method Validation Guidance (2018) emphasises flexibility and a fit-for-purpose approach, requiring stability studies, evaluation of matrix effects, and incurred sample reanalysis, while allowing adaptation to study design [24]. The EMA guideline (2009), more prescriptive in its requirements, has since been superseded by the ICH M10 guideline (2022), which harmonises validation and sample analysis across regions [23,25]. However, ICH M10 still leaves grey areas, particularly with respect to novel sorbents and green solvents, meaning that methods accepted under one framework may not be automatically transferable to another [25]. This regulatory heterogeneity is particularly relevant for miniaturised extraction and microsampling techniques [23,24,25].

**Figure 3:**
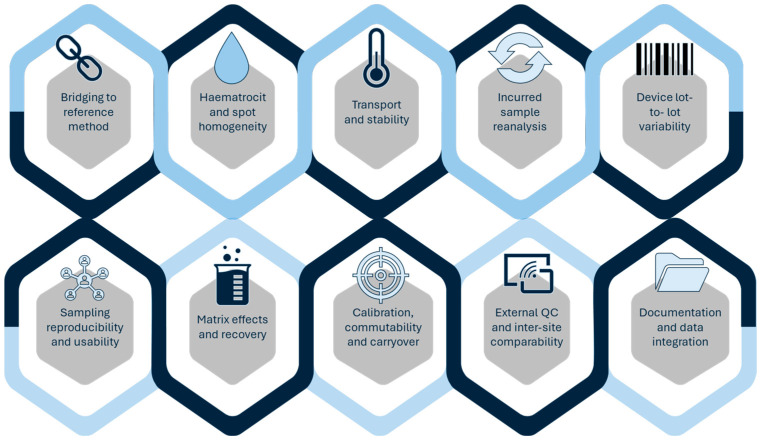
Ten-point validation and translation checklist for microsampling-based TDM.

Validation limitations remain one of the main barriers to clinical translation. DBS, despite its utility in paediatrics and transplantation, is often affected by haematocrit-related bias and insufficient stability testing under long-term storage or variable transport conditions [14,26,30,38]. DPS assays similarly require rigorous evaluation of recovery and matrix effects before replacing conventional plasma-based methods [41,42]. Solvent-based approaches such as DLLME or SALLME, although analytically powerful, are often validated using spiked plasma rather than incurred samples, despite regulatory expectations for incurred sample reanalysis (ISR) to ensure robustness under real-world conditions [29,107,108,109,110,111,112,113,114]. A practical ten-point checklist to support validation and translation of microsampling workflows is provided in Figure 3.

Reproducibility and inter-laboratory harmonisation remain additional bottlenecks. Many studies report strong intra-laboratory performance but lack independent replication, undermining confidence in broader acceptance [6,7,12,21]. For example, MEPS procedures for antipsychotics and antiepileptics have been validated in single-centre workflows [76,77,78,79,80], but external validation is lacking. Similarly, SPME coupled to microfluidic open interfaces has shown excellent sensitivity for tacrolimus and sirolimus, yet no inter-laboratory harmonisation has been demonstrated [86,89,90,91,92]. Device-related variability also poses challenges: differences in absorptive capacity or surface chemistry between batches of VAMS or FPSE devices may affect extraction efficiency, while the absence of certified reference materials prevents robust external quality assurance [8,16,30,75,104].

Another critical issue is the misalignment between analytical validation and clinical applicability. Many methods are optimised for analytical performance but not stress-tested under conditions relevant to personalised medicine, such as home sampling, postal transport, or use in fragile populations [6,8,11,28,30]. VAMS has progressed further, with successful clinical implementation for sirolimus monitoring in paediatric renal transplant recipients and fluconazole in children, supported by validation that incorporated stability, ISR, and bridging with plasma [126,127,128].

In line with the ICH M10 criteria, the degree of regulatory readiness varies considerably among miniaturised extraction techniques. Dried matrix-based formats such as DMS and VAMS generally meet accuracy and precision requirements once validated against conventional plasma methods, with stability often demonstrated under ambient conditions [10,14,23,24,25,28]. However, selectivity and ISR remain critical issues, particularly due to haematocrit bias and uneven analyte distribution [14,23,24,25,28,43]. MEPS and SPME show strong performance in terms of accuracy, precision, and selectivity, largely owing to controlled extraction conditions and direct LC–MS(/MS) coupling. Nonetheless, stability testing is less frequently addressed, and carryover control remains a key regulatory requirement [5,7,12,16,21]. FPSE and DLLME fulfil selectivity and recovery expectations in proof-of-concept studies; however, reproducibility and long-term stability are rarely evaluated, which limits their readiness for regulated bioanalysis [6,8,12,17,19,29]. Among emerging approaches, µSPE and TFME display promising alignment with ICH M10 validation domains but still lack comprehensive inter-laboratory reproducibility data [5,6,12,17,19,21]. Overall, while most miniaturised techniques demonstrate compliance with core validation parameters in individual studies, full regulatory acceptance will depend on the harmonisation of stability, ISR, and cross-matrix equivalence testing across laboratories [6,8,21,23,24,25,28,43].

In contrast, solvent-based protocols (DLLME, SALLME, HF-LPME, EME) largely remain designed for controlled laboratory conditions, with limited adaptation to decentralised workflows or vulnerable populations [29,106,107,108,109,110,111,112,113,114,116]. Pre-analytical variability (e.g., improper drying, contamination, or labelling errors) is seldom incorporated into validation, despite its high relevance outside controlled laboratory environments [14,28,30,38,43]. Validations relying on artificial or pooled matrices may also underestimate the effects of endogenous interferences and polypharmacy, thereby weakening clinical translation [6,9,21,22,43].

Bridging studies with conventional matrices are also insufficient. For regulatory acceptance, equivalence between dried and plasma matrices must be demonstrated [9,10,28]. While some studies have reported strong correlations for antiepileptics and immunosuppressants [26,46], evidence from DPS and bridging studies shows that matrix effects and analyte partitioning can create discrepancies. Accordingly, statistical concordance tools (e.g., Bland–Altman, Passing–Bablok) and clinically relevant ranges are essential [9,10,28,41,42,43]. The reliance on fortified rather than incurred samples further undermines reliability, as it fails to capture active metabolites, comorbidities, and drug–drug interactions common in personalised medicine [3,6,21,22].

Finally, the use of novel sorbents and green solvents introduces regulatory uncertainty. Innovations such as covalent organic frameworks, metal–organic frameworks, hybrid polymers [17,19,131,132,133,134], and the use of deep eutectic solvents in DLLME [106] provide clear analytical and sustainability benefits. Yet, as these materials are not explicitly covered by FDA, EMA, or ICH guidance, laboratories face ambiguity regarding their validation in regulated studies. This regulatory lag slows adoption, even when technical performance is demonstrably superior [23,24,25].

Importantly, several studies have shown that regulatory alignment is achievable. VAMS-based implementations for sirolimus in paediatric renal transplantation and fluconazole in children are examples where regulatory-compliant validation was successfully combined with clinical application [126,127,128].

In summary, although miniaturised extraction techniques hold significant promise, they continue to face regulatory hurdles that limit their widespread adoption. The main challenges include stability testing, matrix comparability, incurred sample reanalysis, reproducibility across laboratories, and validation tailored to clinical scenarios. Bridging studies with conventional matrices and clearer regulatory guidance for innovative sorbents and solvents will be essential to align analytical innovation with FDA, EMA, and ICH expectations. The resolution of these challenges will ultimately determine whether microsampling and solvent-minimised platforms progress beyond proof-of-concept into regulated implementation in therapeutic drug monitoring [5,6,8,13,14,17,19,29,43].

## 5. Future Gaps and Translational Opportunities

The growing interest in miniaturised sampling and extraction techniques reflects the broader shift towards decentralised, patient-centred approaches in clinical bioanalysis [6,7,8,10,21]. While substantial progress has been made in demonstrating their analytical viability, several key gaps must be addressed to ensure full translation into routine TDM and broader clinical contexts [22,28,38].

A major area of development lies in the automation and portability of miniaturised systems. Although many of these techniques are inherently simple and low-volume, their integration into automated workflows remains limited [16,86,89,91]. Achieving high-throughput compatibility without compromising sensitivity or reproducibility is essential, particularly in hospital laboratories where turnaround time is critical [16,21,84]. At the same time, enhanced portability is needed to support home sampling, mobile health units, and resource-limited settings [28,30,31,32,36]. Compact extraction devices, pre-packaged kits, and field-stable reagents are likely to play central roles in expanding the accessibility of TDM to broader patient populations [6,8,10].

In parallel, the design and optimisation of novel functional materials—including sorbents, membranes, monolithic supports, and surface coatings—will be instrumental in improving analyte selectivity, extraction efficiency, and matrix compatibility [17,19,105]. Materials with tunable affinity, enhanced stability, and bioinspired properties offer new opportunities for tackling complex matrices such as oral fluid, dried blood, or sweat [59,93,123,128]. Furthermore, stimuli-responsive and reusable materials may contribute to cost reduction and greener workflows, aligning with global sustainability goals in analytical practice [12,13,29,84].

Another transformative trend is the digital integration of miniaturised bioanalysis, particularly through convergence with biosensors, wearable devices, and mobile health technologies [1,2,31,33]. Linking sample collection with real-time data acquisition, geolocation, or app-based tracking can enhance traceability, sample integrity, and patient adherence [31,32,33,34,35]. Prototype biosensor platforms that combine on-site extraction with electrochemical or optical readouts are already being developed for analytes such as opioids and antiepileptics [2]. However, further validation and clinical testing are required before such systems can support therapeutic decision-making under regulatory frameworks [22,33].

Despite these advances, regulatory acceptance and routine implementation remain significant challenges. Current guidelines were designed for conventional matrices and often do not fully accommodate the specificities of miniaturised approaches [23,24,25]. Progress will depend on supplementary guidance documents or annexes that explicitly address dried and microsampled specimens [28,104]. Collaboration among developers, regulators, and clinical end-users will be needed to establish appropriate validation frameworks, particularly regarding stability, reproducibility, and comparability with established methods [22,23,24,25,28,38]. Certification and standardisation of sampling devices will also be crucial to ensure batch-to-batch consistency and regulatory confidence.

Importantly, translational readiness must become a central priority. Methods should be assessed not only for analytical robustness but also for usability, acceptance by healthcare professionals, and demonstrable impact on patient care. Clinical studies that measure outcomes such as therapeutic response, dose adjustments, and adherence will be key to justifying the replacement of traditional sampling methods in routine practice [11,28,38].

Finally, sustainability and environmental responsibility must be considered. Miniaturised techniques inherently reduce solvent use and biohazardous waste, but device manufacture, disposal, and energy requirements must also be evaluated. Integrating green chemistry principles, promoting recyclable or biodegradable materials, and assessing life-cycle impact will be essential to ensure that technological innovation advances in parallel with environmental stewardship [12,13,29].

In sum, the future of miniaturised bioanalysis for TDM will depend not only on analytical innovation but also on strategic alignment with clinical, regulatory, and societal priorities. A multidimensional approach—integrating materials science, digital health, regulatory science, and clinical validation—will be required to ensure these technologies deliver real-world benefits in precision medicine.

## 6. Conclusions

Miniaturised sampling and extraction techniques offer clear advantages for therapeutic drug monitoring, including reduced sample volumes, simplified procedures, and improved accessibility in both clinical and decentralised settings. These characteristics make them particularly well-suited to personalised medicine and to vulnerable populations where conventional sampling is limited.

However, for widespread adoption, several challenges must be addressed. Validation requirements need to be tailored to the unique features of miniaturised approaches, and differences between current regulatory frameworks highlight the urgent need for harmonisation. Reproducibility, long-term stability, and matrix effects remain critical issues that must be resolved to ensure analytical reliability.

Future developments should prioritise automation, portable device formats, advanced functional materials with enhanced selectivity, and integration with digital health technologies and biosensors. In addition, real-world clinical studies are required to demonstrate tangible improvements in patient outcomes and healthcare efficiency.

By combining innovation with regulatory alignment and clinical validation, miniaturised techniques have the potential to evolve into standard tools for routine bioanalysis and therapeutic drug monitoring. Continued collaboration among researchers, clinicians, industry partners, and regulatory authorities will be essential to realise this goal.

## Figures and Tables

**Table 1:** Examples of DMS techniques in TDM.

Target Drug(s)	Matrix	Type of Paper	Analytical Technique	LOD (ng/mL)	LLOQ/ LOQ (ng/mL)	Linearity (ng/mL)	Application Context	Reference
Phenytoin Lamotrigine Levetiracetam Topiramate Carbamazepine Oxcarbazepine 10,11-dihydro-10-hydroxy carbamazepine	Blood Plasma	Whatman 903^®^ cards Noviplex^®^ plasma prep cards	LC-MS/MS	n.r	0.50 0.50 0.50 0.50 0.25 0.25 0.50 µg/mL	0.50–50 0.50–50 0.50–50 0.50–50 0.25–50 0.25–50 0.50–50 µg/mL	Applied to clinical samples from patients under antiepileptic drug therapy.	[26]
Remifentanil	Blood	Whatman 903^®^ cards	LC-MS/MS	n.r	0.30	0.30–40	Sensitive method for monitoring remifentanil in neonates via non-invasive umbilical cord blood sampling to support efficacy and safety trials.	[27]
Methotrexate	Plasma	Noviplex^®^ plasma prep cards	LC-MS/MS	n.r	30.00	30–2000	Quantification of methotrexate in plasma for potential TDM application.	[41]
Meropenem	Plasma	Noviplex^®^ plasma prep cards	LC-MS/MS	n.r	0.50 µg/mL	0.50–50 µg/mL	DPS method developed for routine TDM of meropenem in clinical laboratory settings.	[42]
Tacrolimus	Blood	hemaPEN^®^ device	LC-MS/MS	n.r	1.00	1.00–100	Adapted for simplified and faster TDM of tacrolimus in transplant patients.	[44]
Vigabatrin Levetiracetam Pregabalin Gabapentin Lamotrigine Lacosamide Zonisamide Rufinamide Topiramate Oxcarbazepine Carbamazepine	Blood	Whatman 903^®^ cards	LC-MS/MS	0.9 0.3 1.4 4.6 1.00 1.4 8.1 0.76 1.00 0.75 0.46	4.80 0.90 4.50 13.90 3.00 4.40 25.00 2.30 4.50 2.30 1.40	5–25,000 1–25,000 5–25,000 14–10,000 3–10,000 5–10,000 25–25,000 2.3–25,000 4.5–10,000 2.3–25,000 1.4–25,000	Demonstrated to be a promising and advanced method for TDM of antiepileptic drugs.	[45]
Abiraterone delta(4)-abiraterone	Blood	Whatman 903^®^ paper cards	LC-MS/MS	n.r	LLOQ: 1 LLOQ: 0.2	1–320 0.2–16	Quantification of Abiraterone and delta(4)-abiraterone in DBS and evaluation of its clinical applicability in patients treated with abiraterone acetate.	[46]
Carbamazepine Lacosamide Lamotrigine Levetiracetam Valproic acid	Blood	Capitainer^®^	UHPLC-MS/MS	0.38 0.48 0.46 0.94 9.67	0.50 0.60 0.60 1.20 120	n.r	Clinically validated using samples from patients with epilepsy.	[47]
Olanzapine Clozapine N-desmethyl clozapine Quetiapine Norquetiapine Aripiprazole Dehydro aripiprazole Chlorpromazine Risperidone 9-hydroxyrisperidone Ziprasidone Perhenazine Fluphenazine Haloperidol Perospirone Amisulpride Sulpiride Memantine Donepezil	Blood	AutoCollect™ DBS cards	LC-MS/MS	0.33 0.71 0.34 0.05 0.04 0.38 0.46 0.16 0.04 0.06 0.25 0.03 0.06 0.01 0.01 0.01 0.19 0.03 0.08	1.11 2.38 1.14 0.17 0.14 1.27 1.53 0.54 0.14 0.22 0.85 0.11 0.20 0.03 0.03 0.03 0.63 0.11 0.27	4–200 40–2000 20–1000 40–2000 20–1000 40–2000 20–1000 16–800 5–250 5–250 16–800 0.2–10 0.6–30 0.6–30 0.6–30 24–1200 40–2000 12–600 4–200	Applied in a fully automated DBS–Online SPE–LC–MS/MS system for the therapeutic drug monitoring of antipsychotic drugs.	[48]
Imatinib Norimatinib	Blood	Ahlstrom 222 discs sheets Capitainer^®^ B device. Whatman 903^®^ cards HemaXis^®^ DB10 device	LC-MS/MS	n.r	240.00 48.00	240–6000 48–1200	Successfully applied to clinical TDM samples using the DMS approach. Supports the use of both devices as practical alternatives for TDM in patients undergoing IMA-based cancer therapy.	[49]
Isavuconazole	Blood	QIAcard FTP DMPK-C cards	HPLC-FLD	n.r	0.10 µg/mL	0.10–20 µg/mL	Demonstrated the viability of DBS as a matrix for TDM of isavuconazole in patients treated with Cresemba^®^.	[50]
Imipenem Meropenem Tigecycline Teclopidine Vancomycin	Blood	Whatman 903^®^ cards	LC-MS/MS	n.r	0.25 0.20 0.016 0.65 0.40 µg/mL	0.25–25 0.2–20 16–1600 0.65–65 0.4–40 µg/mL	Validated as a tool for guiding individualised antimicrobial therapy in ICU patients, suggesting DBS as a complement or alternative to traditional methods.	[51]
Procainamide Lidocaine Quinidine Deslanoside Digoxin Atorvastatin Digitoxin Amiodarone	Blood	Whatman 903^®^ cards	LC-MS/MS	100.00 50.00 100.00 0.10 0.10 0.10 0.50 100.00	500.00 250.00 500.00 0.50 0.50 0.50 1.00 250.00	500–8000 500–5000 2000–6000 0.8–2.4 0.5–2 3–150 10–30 1500–2500	Provides a valuable tool for monitoring cardiovascular therapy in multi-dose regimens.	[52]
Bedaquiline *N*-Desmethylbedaquiline Linezolid Levofloxacin Clofazimine	Blood	Whatman 903^®^ cards	LC-MS/MS	n.r	0.0181 0.00905 0.113 0.0741 0.00814 µg/mL	0.0181–4.94 0.00905–2.47 0.113–30.9 0.0741–20.2 0.00814–2.22 µg/mL	Proposed as a suitable alternative to traditional methods for TDM in remote or resource-limited settings.	[53]
Letrozole Palbociclib Ribociclib Abemaciclib M2 M20	Blood	HemaXis^®^ DB10 device	LC-MS/MS	n.r	6.00 6.00 120.00 40.00 20.00 20.00	6–300 6–300 120–6000 40–800 20–400 20–400	Offers a reliable approach for TDM implementation in hormone receptor-positive, HER2-negative breast cancer, with potential benefits for adherence and outcomes.	[54]
Cabotegravir Rilpivirine	Blood	Whatman 903^®^ cards	LC-MS/MS	n.r	25.00 2.00	25–20,000 2–2500	Applied to quantify analytes from DBS collected during a clinical trial, including post hoc evaluation of paired samples.	[55]
Rifampicin Ethambutol Isoniazid Pyrazinamide	Blood	Whatman 903^®^ cards	UHPLC-MS/MS	0.035 0.021 0.118 0.639 µg/mL	0.42 0.36 0.36 1.98 µg/mL	0.37–24 0.37–24 0.37–24 2.03–130 µg/mL	Demonstrated clinical applicability of bioanalytical methods for TDM purposes.	[56]
Meropenem	Blood	FTA DMPK-B cards	LC-MS/MS	n.r	0.30 µg/mL	0.3–80 µg/mL	Proposed as an alternative strategy for meropenem TDM in preterm neonates, offering adequate performance and logistical benefits.	[57]
Polymyxin B1 Polymyxin B2	Blood	Whatman 903^®^ cards	LC-MS/MS	n.r	0.10 0.0340 µg/mL	0.1–10 0.0110–1.10 µg/mL	Successfully applied in clinical TDM of polymyxin B, representing a feasible alternative for hospital use.	[58]
Clozapine Norclozapine	Plasma Blood	Whatman 903^®^ cards	LC-MS/MS	n.r	50.00	50–1500	Applied to evaluate clozapine therapy in a cohort of schizophrenic patients.	[59]
Bortezomib	Blood	Whatman 903^®^ cards	LC-MS/MS	n.r	0.20	0.2–20	Shows potential for future TDM and pharmacokinetic studies of bortezomib, particularly in paediatric populations.	[60]
Lamotrigine	Blood Oral Fluid	Whatman 903^®^ cards	LC-MS/MS	Blood: 0.01 OF: 0.02 µg/mL	Blood: 1.00 OF:0.50 µg/mL	Blood: 1–30 OF: 0.5–20 µg/mL	Developed to establish a robust protocol for TDM using alternative matrices such as DBS and oral fluid, promoting less invasive sampling.	[61]
Bictegravir	Plasma	Whatman filter paper	LC-MS/MS	n.r	20.00	20–1200	Successfully applied in routine pharmaceutical and industrial studies for sample collection and pharmacokinetic analysis.	[62]
Apixaban	Plasma	n.r	LC-MS/MS	n.r	31.25	31.25–500	Developed to facilitate apixaban TDM, enabling accessibility in peripheral hospitals via DPS shipment.	[63]
Osimertinib AZ5104 AZ7550	Plasma	HemaPEN^®^ device	UHPLC-MS/MS	n.r	1.00	1–729	Useful for managing drug-related toxicity and supporting pharmacokinetic studies in clinical settings.	[64]
Voriconazole Posaconazole Isavuconazole	Plasma	Whatman 903^®^ cards	LC-MS/MS	n.r	0.065 0.22 0.22 µg/mL	0.37–7.74 0.24–496 0.51–20.40 µg/mL	Demonstrated the suitability of DPS for routine TDM of voriconazole and isavuconazole.	[65]
Aciclovir Ganciclovir	Plasma	n.r	LC-MS/MS	n.r	0.013 µg/mL	0.013–20 µg/mL	Adapted for TDM application of antiviral drugs, facilitating dose-concentration assessments, particularly in paediatric patients.	[66]
Fluconazole Posaconazole Itraconazole Hydroxyitraconazole Voriconazole	Plasma	DBS (Whatman grade 31ET chromatography paper)	PS-MS/MS	n.r	0.50 0.10 0.10 0.10 µg/mL	0.5–50 0.1–10 0.1–10 0.1–10 µg/mL	Simultaneous quantitation of five triazole antifungal agents in plasma. The method represents a powerful tool for near-point-of-care TDM, with the potential to improve patient care by reducing turnaround time and supporting clinical research applications.	[67]
Amikacin	Serum	Whatman 903^®^ cards	LC-MS/MS	n.r	0.50 µg/mL	0.5–100 µg/mL	DMS method has been successfully applied in TDM samples	[68]

DBS (dried blood spot), DMS (dried matrix spot), DPS (dried plasma spot), LC (liquid chromatography), HPLC (high-performance liquid chromatography), UHPLC (ultra-high-performance liquid chromatography), GC–MS (gas chromatography–mass spectrometry), PS–MS/MS (paper spray–tandem mass spectrometry), FLD (fluorescence detection), DAD (diode-array detection), SPE (solid-phase extraction), LOD (limit of detection), LOQ (limit of quantification), LLOQ ( lower limit of quantification), MS/MS (tandem mass spectrometry), ICU (intensive care unit), TDM (therapeutic drug monitoring), HER2 (human epidermal growth factor receptor 2), n.r. (not reported).

**Table 2:** Reported applications of MEPS in TDM.

Target Drug(s)	Matrix	Type of Sorbent	Analytical technique	LOD (ng/mL)	LLOQ/LOQ (ng/mL)	Linearity (ng/mL)	Application Context	Reference
Haloperidol Olanzapine Clonazepam Mirtazapine Paroxetine Citalopram Sertraline Chlorpromazine Imipramine Clomipramine Quetiapine Diazepam Fluoxetine Clozapine Carbamazepine Lamotrigine	Plasma	Organic–inorganic hybrid silica monolith	LC-MS/MS	n.r	0.05 0.05 0.10 0.05 0.05 1.00 0.05 0.10 0.05 0.10 0.05 0.05 0.05 0.05 0.10 1.00	0.5–40.5 0.5–40.5 5–155 5–155 5–155 5–325 5–325 10–290 10–290 10–510 10–410 50–850 50–850 50–1550 500–10,500 500–10,500	Used for the quantification of antipsychotic drugs in plasma from schizophrenic patients.	[71]
Nordoxepin Doxepin Desipramine Nortriptyline Imipramine Amitriptyline	Oral fluid	M1	UHPLC-MS	0.04 0.01 0.04 0.01 0.03 0.02	0.13 0.03 0.14 0.03 0.09 0.08	2.0–10.0	Useful tool in clinical and forensic laboratories to quantify TCADs and their metabolites at therapeutic levels.	[72]
Epinephrine Norepinephrine Dopamine Metanephrine Normetanephrine 3-Methoxytyramine	Urine	C_18_	LC-MS/MS	0.08 0.30 0.530 0.176 0.440 0.176	0.167 0.650 1.53 0.440 1.10 0.880	0.167–33.4 0.650–130 1.53–306 1.34–268 3.43–686 1.33–265	Used in routine clinical laboratories for the screening of pheochromocytoma and paraganglioma (PPGL).	[73]
Morphine 6-MAM Cocaine Cocaethylene Benzoylecgonine Methadone EDDP MDPV Mephedrone Methylone Buprenorphine Naloxone Pentedrone Butylone Ethylcathinone Ethylcathinone ephedrine Methylephedrine Pyrovalerone Flephedrone Scopolamine	Oral fluid	M1	UHPLC-MS/MS	2.50 1.00 0.25 0.25 0.50 0.25 0.25 0.25 0.25 0.25 0.25 0.50 0.25 0.25 0.25 0.25 0.25 0.25 0.25 1.00	10.00 2.50 0.50 0.50 1.00 0.50 0.50 0.50 0.50 0.50 0.50 1.00 0.50 0.50 0.50 0.50 0.50 0.50 0.50 2.50	LOQ—250	Applied to patients under substitution therapy programmes and for drug monitoring in traffic safety contexts.	[74]
Chlorpromazine Clozapine Olanzapine Quetiapine	Plasma	RACNT	UHPLC-MS/MS	n.r	10.00 10.00 10.00 10.00	10–700 10–700 10–200 10–700	Applied to the therapeutic drug monitoring of antipsychotic drugs in patients with schizophrenia.	[76]
Ziprasidone	Plasma	C_2_	HPLC-UV	0.30	1.00	1–500	Applied to the therapeutic drug monitoring of psychiatric patients treated with ziprasidone.	[77]
Zonisamide	Plasma	C_18_	HPLC-DAD	n.r	0.20 µg/mL	0.2–80 µg/mL	Applied to plasma samples from epilepsy patients treated with zonisamide to support therapeutic drug monitoring.	[78]
Venlafaxine *O*-desmethylvenlafaxine	Plasma	C_18_	UHPLC-FLD	2.00 5.00	10.00 20.00	10–1000 20–1000	Developed for therapeutic drug monitoring and to support pharmacokinetic studies in humans.	[79]
Amiodarone Desethylamiodarone	Plasma	C_18_	HPLC-DAD	0.02 µg/mL	0.10 µg/mL	0.1–10 µg/mL	Used in patients treated with amiodarone to support TDM and pharmacokinetic studies, such as bioavailability and bioequivalence.	[80]
Cyclosporine A Everolimus Mycophenolic acid Sirolimus Tacrolimus	Serum	C_18_	EC-LC-MS/MS	0.021 0.023 0.027 0.029 0.031	0.063 0.068 0.092 0.098 0.113	1–50	Applied to study the generation of metabolites from selected immunosuppressive drugs.	[81]
Lidocaine Ropivacaine Bupivacaine	Blood	C_18_	LC-MS/MS	n.r	10.00 nmol/L	10–10,000 nmol/L	Used for the therapeutic drug monitoring of target analytes in whole blood.	[82]

RACNT (restricted-access carbon nanotube), C_18_ (octadecyl-bonded silica, reversed-phase sorbent), C_2_ (ethyl-bonded silica), M1 (80% C_8_ and 20% SCX), UHPLC–MS/MS (ultra-high-performance liquid chromatography–tandem mass spectrometry), UHPLC–MS (ultra-high-performance liquid chromatography–mass spectrometry), LC–MS/MS (liquid chromatography–tandem mass spectrometry), LOD (limit of detection), LOQ (limit of quantification), LLOQ ( lower limit of quantification), HPLC (high-performance liquid chromatography), UV (ultraviolet detection), DAD (diode-array detection), FLD (fluorescence detection), EC (electrochemistry), TCADs (tricyclic antidepressants), 6-MAM (6-monoacetylmorphine), EDDP (2-ethylidene-1,5-dimethyl-3,3-diphenylpyrrolidine), and MDPV (3,4-methylenedioxypyrovalerone), PPGL (pheochromocytoma and paraganglioma), n.r (not reported).

**Table 3:** Use of SPME for therapeutic drug quantification.

Target Drug(s)	Matrix	Type of Fiber	Analytical Technique	LOD (ng/mL)	LLOQ/LOQ (ng/mL)	Linearity (ng/mL)	Application Context	Reference
Amitriptyline Aripiprazole Carbamazepine Citalopram Clomipramine Desipramine Flunitrazepam Fluoxetine Fluvoxamine Imipramine Ketamine Paroxetine Sertraline Trazodone Duloxetine Mirtazapine Nortriptyline Venlafaxine Lamotrigine Quetiapine Olanzapine	Blood	C-18 SPME-LC silica	LC-TOF-MS	0.18 n.r n.r 0.97 n.r n.r n.r 0.46 n.r n.r n.r 0.52 1.69 0.54 4.29 0.14 n.r 0.58 0.37 0.92 1.52	0.92 n.r n.r 4.87 n.r n.r n.r 2.32 n.r n.r n.r 2.60 8.43 2.69 21.47 0.70 n.r 2.91 5.73 54.58 7.62	LLOQ—300	DI-SPME applied to 38 blood samples from patients with mood disorders; enabled rapid screening of drug concentrations within therapeutic and toxic dose ranges.	[83]
Tacrolimus Sirolimus Everolimus Cyclosporine A	Blood	Bio-SPME fibers coated with HLB particles	MOI-MS/MS	0.3 0.2 0.3 0.3	0.80 0.70 1.00 0.80	1–50 1–50 1–50 2.5–500	Applied for the determination of immunosuppressive drug levels in whole blood samples.	[86]
Gefitinib O-desmethyl-gefitinib	Plasma	SPMELC C18 tips	LC-MS/MS	n.r	20.00 20.00	20–800	Applied for proof-of-concept analysis of plasma samples from individuals receiving gefitinib treatment.	[87]
Imatinib N-desmethylimatinib	Plasma	hollow fiber (polysulfone, polyvinyl chloride, polyacrylonitrile)	LC-MS/MS	0.50 1.00	2.50 2.50	2.5–250	Successfully implemented in clinical TDM for imatinib and norimatinib; both total and unbound concentrations were evaluated in clinical samples.	[88]
Tranexamic acid	Plasma	Nitinol coated with HLB particles	MOI-MS/MS	n.r	25.00 µg/mL	25–2000 µg/mL	Enabled near real-time monitoring of tranexamic acid concentrations in plasma during surgical procedures.	[89]
Levodopa Carbidopa Benserazide Dopamine 3-O-methyldopa	Plasma	aminopropyl hybrid silica monolith containing SBA-15	HILIC-MS/MS	n.r	22.00 33.00 170.00 1.20 10.00	22–2000 33–2000 170–2000 1.2–2000 10–2000	Applied to plasma samples from patients with Parkinson’s disease for routine TDM.	[90]
Atenolol Morphine Acetaminophen Lorazepam Carbamazepine Diazepam Buprenorphine	Plasma	BioSPME (C18-bonded silica particles in combination with biocompatible polyacrylonitrile (PAN) polymeric adhesive)	LC-MS/MS	n.r	n.r	n.r	Used in high-throughput platforms to determine free plasma concentrations and protein binding of drugs with diverse physicochemical profiles.	[91]
Tranexamic acid	Urine Plasma	PAN and HLB particles	LC-MS/MS	n.r	Urine: 25.00 Plasma: 10.00 µg/mL	Urine: 25–1000 µg/mL Plasma: 10–1000 µg/mL	Developed an improved SPME-based sampling protocol for TDM of tranexamic acid in plasma and urine from patients with chronic renal dysfunction, aiming to optimise dosing regimens.	[92]
Caffeine	Serum Oral Fluid	ZB-FFAP, with 100% nitroterephthalic modified polyethylene glycol	HPLC-DAD	n.r	n.r	n.r	Enabled OF-based quantification of caffeine in preterm infants; showed strong correlation with serum levels during treatment for apnoea of prematurity.	[93]
Valproic acid	Serum	BioSPME (LC Tips C18)	GC-MS	n.r	10.00 µg/mL	10–150 µg/mL	Developed and validated a GC–MS assay using BioSPME for the quantification of valproic acid in serum, supporting routine TDM applications.	[94]
Amitriptyline Doxepin Nortriptyline	Serum Liver Kidney Brain	COF	ESI-MS	0.10 0.50 0.10	0.30 1.50 0.30	0.8–100 1.0–100 0.8–100	Demonstrated as a powerful analytical tool for therapeutic drug monitoring in clinical settings.	[95]
Doxorubicin Doxorubicinol Doxorubicinone Doxorubicinolone	Lung	BioSPME (mixed-mode fibres with C8+benzenesulfonic acid particle)	LC-MS/MS	n.r	n.r	10–1000 1–100 1–100 1–100 µg/mL	Enabled simultaneous monitoring of drug levels and biodistribution in lung tissue alongside relevant metabolites, providing insights into drug activity and therapeutic optimisation.	[96]

BioSPME (biocompatible solid-phase microextraction), C_18_ (octadecyl-bonded silica; reversed-phase sorbent), C_8_ (octyl-bonded silica; mixed-mode in the cited fibres), COF (covalent organic framework), DI-SPME (direct-immersion solid-phase microextraction), ESI–MS (electrospray ionisation–mass spectrometry), GC–MS (gas chromatography–mass spectrometry), HF (hollow fibre), HILIC–MS/MS (hydrophilic interaction liquid chromatography–tandem mass spectrometry), HLB (hydrophilic–lipophilic balance polymer), DAD (diode-array detection), LC–MS/MS (liquid chromatography–tandem mass spectrometry), LC–TOF–MS (liquid chromatography–time-of-flight mass spectrometry), LOD (limit of detection), LOQ (limit of quantification), LLOQ ( lower limit of quantification), MOI–MS/MS (microfluidic open interface–tandem mass spectrometry), n.r (not reported), OF (oral fluid), PAN (polyacrylonitrile), SBA-15 (Santa Barbara Amorphous-15 mesoporous silica), SPME (solid-phase microextraction), TDM (therapeutic drug monitoring), UHPLC–MS (ultra-high-performance liquid chromatography–mass spectrometry), UHPLC–MS/MS (ultra-high-performance liquid chromatography–tandem mass spectrometry).

**Table 4:** Reported use of FPSE in bioanalytical/TDM applications.

Target Drug(s)	Matrix	Type of Membrane	Analytical Technique	LOD (ng/mL)	LLOQ/LOQ (ng/mL)	Linearity (ng/mL)	Application Context	Reference
Ciprofloxacin Sulfasalazine Cortisone	Blood Plasma Urine	sol–gel Carbowax^®^ 20 M	HPLC-DAD	Blood: 0.02 Plasma: 0.10 Urine: 0.03 µg/mL	Blood: 0.05 Plasma: 0.25 Urine: 0.10 µg/mL	Blood:0.05–10 Plasma: 0.25–10 Urine: 0.10–10 µg/mL	Quantification of therapeutic agents used in the treatment of inflammatory bowel disease.	[97]
BP-4 4-DHB DHMB BP-1 BP-2 benzophenone	Blood Plasma Urine	sol–gel Carbowax^®^ 20 M coating on cellulose	HPLC-PDA	0.03 µg/mL	0.10 µg/mL	0.1–10 µg/mL	Demonstrates utility as a valuable tool for assessing bioaccumulation in humans.	[98]
Pioglitazone Repaglinide Nateglinide	Plasma	sol–gel Carbowax^®^ 20 M with cellulose	HILIC-MS	7.00 2.00 30.00	25.00 6.00 125.00	25–200 6.25–500 125–10,000	Bioanalytical method for monitoring the therapeutic levels of antidiabetic drugs.	[99]
Febuxostat Montelukast	Plasma	sol–gel PCAP-PDMS-PCAP	HPLC-FLD	0.10 1.50	0.30 5.00	0.3–10 5–100	Evaluation of the pharmacokinetic profiles of febuxostat and montelukast in human plasma.	[100]
Favipiravir	Plasma Breast milk	sol–gel PCAP-PDMS-PCAP	HPLC-UV	Plasma: 0.06 Breast milk: 0.15 µg/mL	Plasma: 0.20 Breast milk: 0.50 µg/mL	Plasma: 0.2–50 Breast milk: 0.5–25 µg/mL	Quantification of favipiravir in plasma and breast milk to assess exposure and safety, particularly in the context of limited data in infants.	[101]
Venlafaxine Citalopram Paroxetine Fluoxetine Sertraline Amitriptyline Clomipramine	Blood Urine Oral fluid	sol–gel Carbowax^®^ 20 M coated with cellulose	HPLC-PDA	0.06 0.04 0.04 0.04 0.04 0.04 0.04 mg/mL	0.20 0.10 0.10 0.10 0.10 0.10 0.10 mg/mL	0.2–20 0.1–20 0.1–20 0.1–20 0.1–20 0.1–20 0.1–20 mg/mL	Method developed to optimise pharmacotherapy and enable TDM.	[102]
Fingolimod Citalopram	Artificial oral fluid; Artificial urine	Sol–Gel-PCAP-PDMS-PCAP	HPLC-DAD	7.46 5.97	24.38 19.78	25–1000 20–1000	Determination of two drugs commonly prescribed for the management of multiple sclerosis.	[103]

4-DHB (4-dihydroxybenzophenone), BP-1 (benzophenone-1), BP-2 (benzophenone-2), BP-4 (benzophenone-4), DHMB (dihydroxy-methoxybenzophenone), HILIC–MS (hydrophilic-interaction liquid chromatography–mass spectrometry), DAD (diode-array detection), FLD (fluorescence detection), LOD (limit of detection), LOQ (limit of quantification), LLOQ ( lower limit of quantification), PDA (photodiode-array detection; equivalent to DAD), UV (ultraviolet detection), PCAP (polycaprolactone-based sol–gel precursor), PDMS (polydimethylsiloxane), TDM (therapeutic drug monitoring), Carbowax^®^ 20M refers to a polyethylene-glycol (PEG 20 000) sol–gel coating on cellulose, n.r (not reported).

**Table 5:** Applications of DLLME in therapeutic drug analysis.

Target Drug(s)	Matrix	Type of Solvents	Analytical Technique	LOD (ng/mL)	LLOQ/LOQ (ng/mL)	Linearity (ng/mL)	Application Context	Reference
Amitriptyline hydrochloride Imipramine hydrochloride Sertraline hydrochloride Fluoxetine hydrochloride	Plasma Urine	DES-DDLME-MPAR (Methyltrioctylammonium bromide: capryl alcohol (1:3, *v*/*v*)	HPLC-UV	0.027 0.028 0.025 0.020 µg/mL	0.075 0.092 0.080 0.068 µg/mL	0.1–10 µg/mL	Enabled the simultaneous quantification of selected antidepressants in plasma and urine, supporting pharmacotherapy control and drug toxicity prevention.	[106]
Efavirenz	Plasma	DLLME (acetonitrile: chloroform)	GC-MS	0.008 µg/mL	0.027 µg/mL	0.10–2.0 µg/mL	Applied to human plasma samples from patients undergoing treatment with efavirenz.	[107]
Risperidone 9-hydroxyrisperidone	Plasma	DLLME (chlorobenzene: acetone)	LC-MS/MS	n.r	5.00	5.0–80	Successfully used in plasma analysis of a schizophrenic patient under therapy.	[108]
Tamoxifen *N*-desmethyltamoxifen 4-hydroxytamoxifen Endoxifen	Plasma	DLLME (DES: thymol-nonanoic acid)	HPLC-DAD	1.70 3.20 0.30 0.70	5.00 10.00 0.80 2.00	5–300 10–500 0.8–25 2–50	Effective for pharmacokinetic, pharmacodynamic, and therapeutic drug monitoring studies of tamoxifen and its main metabolites in biological fluids.	[109]
Carbamazepine Phenobarbital	Plasma	DLLME (n.r)	HPLC-UV	n.r	2.00 10.00 µg/mL	n.r	Demonstrates clinical value of plasma-level monitoring for optimising treatment selection and staging intoxication cases.	[110]
Docetaxel	Plasma Urine	UA-DLLME (chloroform: ethanol)	LC-MS/MS	Plasma: 1.00 Urine: 2.50 µg/mL	Plasma: 2.50 Urine: 5.00~ µg/mL	Plasma: 2.5–2000 Urine: 5–2000 µg/mL	Contributed to improved oncological strategies, particularly in paediatric oncology units, by supporting individualised TDM.	[111]
Letrozole Anastrozole Palbociclib Ribociclib Abemaciclib Fulvestrant	Plasma	DLLME (isopropanol, chloroform (1:2, *v*/*v*))	MECK-MS/MS	n.r	30.00 20.00 30.00 500.00 50.00 10.00	30–300 20–200 30–300 500–5000 50–500 10–100	Applied to plasma samples from breast cancer patients; determined concentrations were consistent with clinical data, confirming its suitability for TDM.	[112]
Chlorpromazine	Plasma	DLLME (n.a)	GC-MS	n.r	30.00	30–600	Applied to plasma samples from real patients receiving clinical treatment.	[113]
Abemaciclib Palbociclib Ribociclib Anastrozole Letrozole Fulvestrant	Plasma	DLLME (isopropanol, chloroform)	HPLC-FLD HPLC-DAD	n.r	0.11 0.08 0.25 2.51 0.04 0.50 µg/mL	0.11–2.61 0.08–1.92 0.25–5.95 2.51–60.30 0.04–1.01 0.50–12.04 µg/mL	Successfully used for drug monitoring in breast cancer patient plasma samples.	[114]
Sirolimus	Blood	DLLME (ethanol: chloroform)	LC-MS/MS	0.20	0.60	1–50	Applied for the therapeutic drug monitoring of sirolimus in whole blood samples to assess its pharmacokinetic profile in paediatric patients with lymphatic anomalies at hourly intervals	[115]

DES (deep eutectic solvent), DES–DLLME–MPAR (deep eutectic solvent–dispersive liquid–liquid microextraction with magnetic particles–assisted retrieval), DLLME (dispersive liquid–liquid microextraction), GC–MS (gas chromatography–mass spectrometry), HPLC (high performance liquid chromatography), DAD (diode-array detection), FLD (fluorescence detection), UV (ultraviolet detection), LC–MS/MS (liquid chromatography–tandem mass spectrometry), LOD (limit of detection), LOQ (limit of quantification), LLOQ ( lower limit of quantification), MEKC–MS/MS (micellar electrokinetic capillary chromatography–tandem mass spectrometry; shown in the table as “MECK-MS/MS”), UA–DLLME (ultrasound-assisted DLLME), TDM (therapeutic drug monitoring), n.a. (not available) and n.r. (not reported).

**Table 6:** Emerging miniaturised extraction approaches for drug monitoring.

Target Drug(s)	Matrix	Extraction	Analytical Technique	LOD (ng/mL)	LLOQ/LOQ (ng/mL)	Linearity (ng/mL)	Application Context	Reference
Vancomycin	Plasma	µ-SPE (Oasis^®^ MAX μElution Plate)	UHPLC-MS/MS	n.r	0.50	0.50–100	Successfully applied to the TDM of vancomycin in clinical samples.	[118]
Lamotrigine	Serum	µ-SPE (HLB)	SERS	3.20 µM	9.50 µM	9.5–75 µM	Demonstrates high potential for automation in a compact device suitable for routine clinical settings, as an alternative analytical TDM method for lamotrigine.	[119]
Cannabidiol Tetrahydrocannabinol	Plasma	PT-µSPE (octyl-functionalized hybrid silica monolith)	UHPLC-MS/MS	n.r	10.00	10–150	Demonstrated to be suitable for the simultaneous quantification of cannabidiol and tetrahydrocannabinol in plasma for TDM in patients undergoing cannabinoid-based therapy.	[120]
Venlafaxine Citalopram Imipramine Amitriptyline Sertraline Fluoxetine	Urine; Plasma	PT-µSPE (melt-blown polypropylene nonwoven material)	HPLC-UV	5.70 5.50 4.20 4.20 3.10 3.00	19.10 18.30 14.30 14.10 10.30 10.00	20–200	Offers a practical, scalable, and environmentally friendly approach for therapeutic drug monitoring in clinical settings.	[121]
6-MAM Methamphetamine MDMA Ketamine Norketamine Cocaine	Urine	PT-SPE (C_18_)	miniMS	1.00 1.00 1.00 0.50 1.00 0.25	5.00 5.00 5.00 1.00 5.00 1.00	5–250 5–250 5–250 1–250 5–250 1–250	Enables rapid on-site detection with improved portability and lower cost, supporting its potential use in forensic analysis and drug crime investigation.	[122]
Amphetamine Methamphetamine MDMA MDA MDEA Cocaine Cocaethylene AEME Dibutylone *N*-ethylpentylone 25E-NBOMe 25C-NBOMe 2C-E 2C-C Fentanyl Carfentanil	Sweat	DPX (SCX)	GC-MS	1.00 2.00 1.00 1.00 3.00 5.00 5.00 5.00 1.00 1.00 5.00 3.00 15.00 15.00 3.00 3.00	2.00 3.00 2.00 2.00 5.00 10.00 10.00 10.00 2.00 2.00 10.00 5.00 30.00 30.00 5.00 5.00	LOQ—1100	Demonstrated the utility of sweat as a versatile biofluid for toxicological and forensic applications across clinical, occupational, and legal settings.	[123]
Tamoxifen *N*-desmethyltamoxifen 4-hydroxytamoxifenendoxifen	Plasma	dSPE (Fe_3_O_4_@SiO_2_)	HPLC-DAD	1.50 1.90 0.10 0.30	5.00 5.00 0.20 1.00	5–100 5–200 0.2–25 1–50	Enabled TDM and pharmacodynamic assessment of tamoxifen and metabolites in biological fluids, supporting clinical drug research and therapeutic individualisation.	[124]
Epirubicin	Plasma Urine	m-µSPE (Fe_3_O_4_-based nanoparticles coated with silica and a double-chain surfactant—namely, didodecyldimethylammonium bromide (DDAB))	LC-FLD	0.50 µg/mL	1.00 µg/mL	Plasma: 0.001–1 Urine: 0.001–10 µg/mL	Validated for routine monitoring in clinical practice, enhancing precision medicine approaches.	[125]
Fluconazole	Blood, plasma, serum	VAMS (Mitra^®^ devices)	HPLC-UV	n.r	5.00 µg/mL	5–160 µg/mL	Validated for the monitoring of fluconazole exposure in immunocompromised paediatric patients, with potential applicability to pharmacokinetic studies.	[126]
Sirolimus	Blood	VAMS (Mitra™ device)	LC-MS/MS	0.10	0.35	0.25–60	Implemented in routine clinical practice in paediatric transplant centres, particularly for renal transplant recipients, demonstrating its utility in real-world scenarios.	[127]
Sertraline Fluoxetine Citalopram Vortioxetine Norsertraline Norfluoxetine *N*-desmethylcitalopram *N, N*-didesmethylcitalopram	Blood Oral Fluid	VAMS (Mitra^®^ device)	HPLC-UV-FLD	OF, Blood: 1.50; 2.50 2.50; 3.00 0.30; 0.30 1.00; 1.50 1.50; 2.50 2.50; 3.00 0.30; 0.30 0.30; 0.30	OF, Blood: 5.00; 7.00 7.00; 10.00 1.00; 1.00 3.00; 5.00 5.00; 7.00 7.00; 10.00 1.00; 1.00 1.00;1.00	LOQ—500 LOQ—750 LOQ—200 LOQ—500 LOQ—500 LOQ—750 LOQ—200 LOQ—200	Demonstrates clinical applicability via VAMS-based TDM of antidepressants in blood and oral fluid from psychiatric patients.	[128]
Biperiden	Plasma	SALLME (sodium chloride)	GC-MS	n.r	0.50	0.5–15	Successfully applied to the therapeutic drug monitoring of biperiden in plasma samples from real patients	[129]
Buspirone Alprazolam Clonazepam Diazepam Lorazepam	Plasma	SALLME (zinc sulphate)	LC-MS/MS	n.r	1.00 5.00 4.00 100.00 30.00	1–30 5–100 4–100 100–3000 30–300	Demonstrated suitability for therapeutic drug monitoring through the determination of drug concentrations in real patient samples.	[130]
Ketoprofen Fenbufen Carprofen Diclofenac Ibuprofen	Serum	Chip-based capillary array	HPLC-UV	4.39 8.06 5.40 7.96 15.5	20.00 20.00 20.00 20.00 50.00	20–1000 20–1000 20–1000 20–1000 50–1000	Demonstrated potential for use in TDM, forensic toxicology, and clinical pharmacokinetic investigations.	[131]
Mycophenolic acid	Plasma	TFME (MIP)	UPLC-MS/MS	0.30	1.00	5–250	Demonstrated suitability for therapeutic drug monitoring applications.	[132]
Dasatinib Erlotinib	Plasma	TFME (PLA)	HPLC-DAD	0.03 0.30	0.10 1.00	0.1–20 1–500	Utilised for the quantification of anticancer drugs in human plasma; supports TDM due to the toxicity profile of tyrosine kinase inhibitors (TKIs).	[133]
Piperacilin Imipenem	Blood	TFME (DVB)	LC-MS/MS	n.r	0.02 0.05 µg/mL	0.01–1 µg/mL	Provided a high-throughput analytical platform demonstrating low antibiotic bioavailability at infection sites, enabling personalised dosing strategies through future TDM studies.	[134]
Phenytoin	Plasma	EME (PP Q3/2 polypropylene hollow fiber)	CE-DAD	0.005 µg/mL	0.03 µg/mL	0.03–4 µg/mL	Determination of free phenytoin concentration offers a viable alternative to conventional methods for therapeutic drug monitoring and pharmacokinetic studies.	[135]
Fluoxetine	Serum	HF-LPME (polypropylene)	nanoLC-HRMS	n.r	0.2 µg/mL	0.2–2.5 µg/mL	Demonstrated potential for therapeutic drug monitoring using a lab-fabricated chromatographic nanocolumn.	[136]
Imipramine Desipramine Fluoxetine Norfluoxetine Paroxetine Maprotiline Sertraline Citalopram Clomipramine Trazodone Doxepin Clozapine Amitriptyline	Blood	SLE (Isolute SLE + cartridge)	UPLC-MS/MS	0.03 0.0003 0.0003 0.003 0.003 0.0003 0.0003 0.0003 0.0003 0.0003 0.003 0.0003 0.0003	0.01 0.001 0.001 0.01 0.01 0.001 0.001 0.001 0.001 0.001 0.01 0.001 0.001	0.01–200 0.001–200 0.001–200 0.01–200 0.01–200 0.001–200 0.001–200 0.001–200 0.001–200 0.001–200 0.01–200 0.001–200 0.001–200	Applied to real-case analysis of antidepressants, demonstrating potential for broad use in both biomedical and forensic contexts.	[137]
Amitriptyline Carbamazepine Citalopram Clomipramine Clonazepam Codeine Diazepam Flunitrazepam Fluoxetine Flurazepam Norfluoxetine Nortriptyline Paroxetine Sertraline Venlafaxine	Blood	mini-QuEchERS (magnesium sulphate, sodium chloride and sodium citrate dihydrate (4:1:1, *w*/*w*/*w*))	UPLC-MS/MS	n.r	25.00	25–1000	Demonstrates capability for the determination of target psychotropic drugs and metabolites in forensic and clinical toxicology.	[138]

CE–DAD (capillary electrophoresis with diode-array detection), dSPE (dispersive solid-phase extraction), DPX (dispersive pipette extraction), DVB (divinylbenzene), EME (electromembrane extraction), Fe_3_O_4_@SiO_2_ (magnetite@silica core–shell nanoparticles), GC–MS (gas chromatography–mass spectrometry), HF–LPME (hollow-fibre liquid-phase microextraction), HLB (hydrophilic–lipophilic balance polymer), HPLC–DAD (high-performance liquid chromatography with diode-array detection), UV (ultraviolet detection), FLD (fluorescence detection), HRMS (high-resolution mass spectrometry),), IL (ionic liquid), FF (Ferrofluids), LC (liquid chromatography), LC–MS/MS (liquid chromatography–tandem mass spectrometry), LOD (limit of detection), LOQ (limit of quantification), LLOQ ( lower limit of quantification), mini-QuEChERS (a scaled QuEChERS procedure; Quick, Easy, Cheap, Effective, Rugged and Safe), MS (mass spectrometer), MIP (molecularly imprinted polymer), m-µSPE (magnetic micro-solid-phase extraction), PLA (poly(lactic acid)), PP (polypropylene), PT–µSPE (pipette-tip micro-solid-phase extraction), PT–SPE (pipette-tip solid-phase extraction), SALLME (salt-assisted liquid–liquid microextraction), SERS (surface-enhanced Raman scattering), SLE (supported liquid extraction), SCX (strong cation exchange), TFME (thin-film microextraction), TDM (therapeutic drug monitoring), TKIs (tyrosine kinase inhibitors), UPLC–MS/MS (ultra-performance liquid chromatography–tandem mass spectrometry), UHPLC–MS/MS (ultra-high-performance liquid chromatography–tandem mass spectrometry), VAMS (volumetric absorptive microsampling), µ-SPE (micro-solid-phase extraction), UA (Ultrasound-Assisted).
